# Varus tibial alignment is associated with improved joint awareness after lateral unicompartmental knee arthroplasty

**DOI:** 10.1002/jeo2.70516

**Published:** 2025-11-05

**Authors:** Mustafa Hariri, Paul Mick, Johannes Weishorn, Timo A. Nees, Kevin‐Arno Koch, Tobias Reiner, Tilman Walker, Raphael Trefzer

**Affiliations:** ^1^ Department of Orthopaedics Heidelberg University Hospital Heidelberg Germany

**Keywords:** clinical outcome, component alignment, lateral UKA, radiographic analysis, unicompartmental knee replacement

## Abstract

**Purpose:**

Due to anatomic and kinematic differences between the medial and lateral knee compartments, findings from medial unicompartmental knee arthroplasty (UKA) cannot be directly applied to lateral UKA. In particular, optimal component alignment may differ between both compartments. Therefore, the study aimed to assess the potential association of femoral and tibial component alignment on clinical outcomes in patients undergoing lateral UKA and to determine whether recommendations for optimal component positioning can be derived from the findings.

**Methods:**

This retrospective study analysed prospectively collected data from patients who underwent lateral UKA with a fixed‐bearing implant. Radiographic alignment parameters were measured relative to the anatomical axes of the femur and tibia, including femoral varus/valgus angle (VVA‐F), tibial varus/valgus angle (VVA‐T), femoral flexion/extension angle (FEA‐F) and tibial slope angle (TSA). Clinical outcomes were assessed using the Oxford Knee Score, Forgotten Joint Score (FJS) and range of motion. Statistical analyses included correlation and subgroup analyses to identify relationships between component alignment and clinical outcomes.

**Results:**

A total of 94 patients (97 UKA) were eligible for clinical and radiological analysis with a mean follow‐up of 3.4 ± 1.9 years. Most component alignment variations did not significantly affect clinical outcomes. However, a varus‐aligned tibial component correlated with an improved FJS (*p* = 0.021). Based on the findings, target alignment ranges for lateral UKA were proposed as safe thresholds: VVA‐F: 3° varus to 13° valgus, VVA‐T: 3° varus to 1° valgus, FEA‐F: 3.5° extension to 16.5° flexion and TSA: 4.5°–10.5°.

**Conclusion:**

Although previous studies have addressed component alignment in UKA, evidence specifically related to lateral fixed‐bearing UKA remains limited. The present study contributes to this underexplored area by providing preliminary data suggesting that slight varus alignment of the tibial component may be associated with improved clinical outcomes in lateral UKA.

**Level of Evidence:**

Level III, retrospective cohort study.

**Abbreviations**
AP: anteroposterior
BMI: body mass index
FEA‐F: flexion/extension angle of the femoral component
FJS: forgotten Joint Score
HKA: hip–knee–ankle angle
ICC: intraclass correlation coefficients
OA: osteoarthritis
OKS: Oxford Knee Score
ROM: range of motion
SD: standard deviation
TKA: total knee arthroplasty
TSA: tibial slope angle
UKA: unicompartmental knee arthroplasty
VVA‐F: varus/valgus angle of the femoral component
VVA‐T: varus/valgus angle of the tibial component

## INTRODUCTION

Unicompartmental knee arthroplasty (UKA) is a well‐established surgical intervention for managing unicompartmental osteoarthritis (OA), offering benefits such as faster recovery, improved functional outcome and fewer adverse events compared to total knee arthroplasty (TKA) [20, 23, 32]. While extensive research has been conducted on medial UKA, the lateral compartment has received comparatively less attention due to its significantly lower prevalence [37]. Nevertheless, research over the past decade has demonstrated the superiority of a fixed‐bearing design in lateral UKA due to persistently high rates of bearing dislocation in mobile‐bearing designs [7, 11]. The contrasting findings compared to medial UKA, where both designs perform well, can be explained by biomechanical and anatomical differences between the medial and lateral compartments [42]. These include greater distraction of the lateral compartment and a more pronounced posterior movement of the lateral femoral condyle in deep flexion [30, 34, 44].

Proper alignment of femoral and tibial components is crucial for the success of knee arthroplasty procedures and malalignment may lead to increased wear and implant loosening, particularly in fixed‐bearing implants, where edge‐loading is more relevant [1, 15, 19, 22, 39]. In medial UKA, some studies have investigated the influence of component positioning on postoperative outcomes, demonstrating no significant association with clinical results [5, 9, 10, 17]. However, the lateral compartment exhibits unique kinematic characteristics, making it inappropriate to directly apply medial UKA alignment principles to the lateral side.

While a few studies have investigated the influence of the hip–knee–ankle angle (HKA) in lateral UKA and reported favourable clinical outcomes in patients with moderate valgus alignment, there is currently no established recommendation regarding acceptable component alignment ranges for lateral UKA [26, 27].

Therefore, the objective of this exploratory study is twofold: first, to assess the potential impact of femoral and tibial component alignment on clinical outcomes in patients undergoing lateral UKA with a fixed‐bearing implant and second, to determine whether recommendations for optimal component positioning can be derived from the findings. It was hypothesised that component alignment within a defined range would not be significantly associated with most short‐ to mid‐term clinical outcomes, but that specific alignment parameters might influence certain aspects of postoperative knee function and patient‐reported outcomes.

## METHODS

This retrospective cohort study based on prospectively collected data and was approved by the Institutional Review Board of the University of Heidelberg (S‐079/2023). Informed consent was provided by all patients and the study adhered to the 2013 revision of the Helsinki Declaration.

### Inclusion and exclusion criteria

Patients older than 18 years with isolated lateral compartment OA who underwent lateral UKA with a fixed‐bearing implant (Oxford Fixed Lateral, Biomet Inc., Warsaw, Indiana) at one single institution between 2013 and 2020 were included. A minimum follow‐up of 12 months was required. Data were retrieved from the prospectively maintained institutional arthroplasty database. Patients with previous corrective osteotomy, simultaneous surgical procedures, or incomplete follow‐up were excluded.

### Indication, surgical technique and rehabilitation

Indications included severe lateral compartment OA with full‐thickness cartilage loss (bone‐on‐bone) or femoral condyle avascular necrosis. Functional integrity of anterior cruciate ligament, medial collateral ligament, and lateral collateral ligament was confirmed clinically, with correctable valgus deformity and absence of medial compartment OA on varus stress radiographs. Patellofemoral OA was not contraindicated unless severe medial facet damage was evident. Rheumatoid arthritis, fixed valgus deformity, or flexion deformity > 15° were contraindications. No simultaneous surgical procedures were performed.

All surgeries utilised a minimally invasive lateral parapatellar approach without patellar dislocation. Tibial plateau internal rotation and anatomical femoral component positioning were ensured to avoid joint line elevation, with bearing thickness selected in full extension. Femoral fixation was cemented or uncemented based on bone quality; tibial components were cemented. Procedures were conducted or supervised by six experienced knee arthroplasty surgeons. Immediate postoperative full weight‐bearing was permitted, with unrestricted knee movement. Rehabilitation lasted approximately three weeks (inpatient or outpatient).

### Data collection

Clinical data were obtained through clinical examination by two of the authors (MH and RT) as part of a regular check‐up to determine the Oxford Knee Score (OKS), the Forgotten Joint Score (FJS) such as the range of motion (ROM). OKS and ROM were evaluated pre‐ and postoperatively, whereas the FJS was assessed only postoperatively [3, 29]. These regular check‐ups are routinely performed for all patients receiving an arthroplasty at our institution at 1‐, 3‐, and 5‐year follow‐up intervals, and include standard anteroposterior (AP) and lateral knee radiographs. These radiographs of the knee were aligned with fluoroscopic control to obtain views parallel to the tibial component in the AP view and parallel to the femoral component in the lateral view. Radiographs were included if they provided sufficient visualisation of the femoral and tibial shaft to allow determination of the anatomical axes. No radiographs had to be excluded for this reason.

**FIGURE 1:**
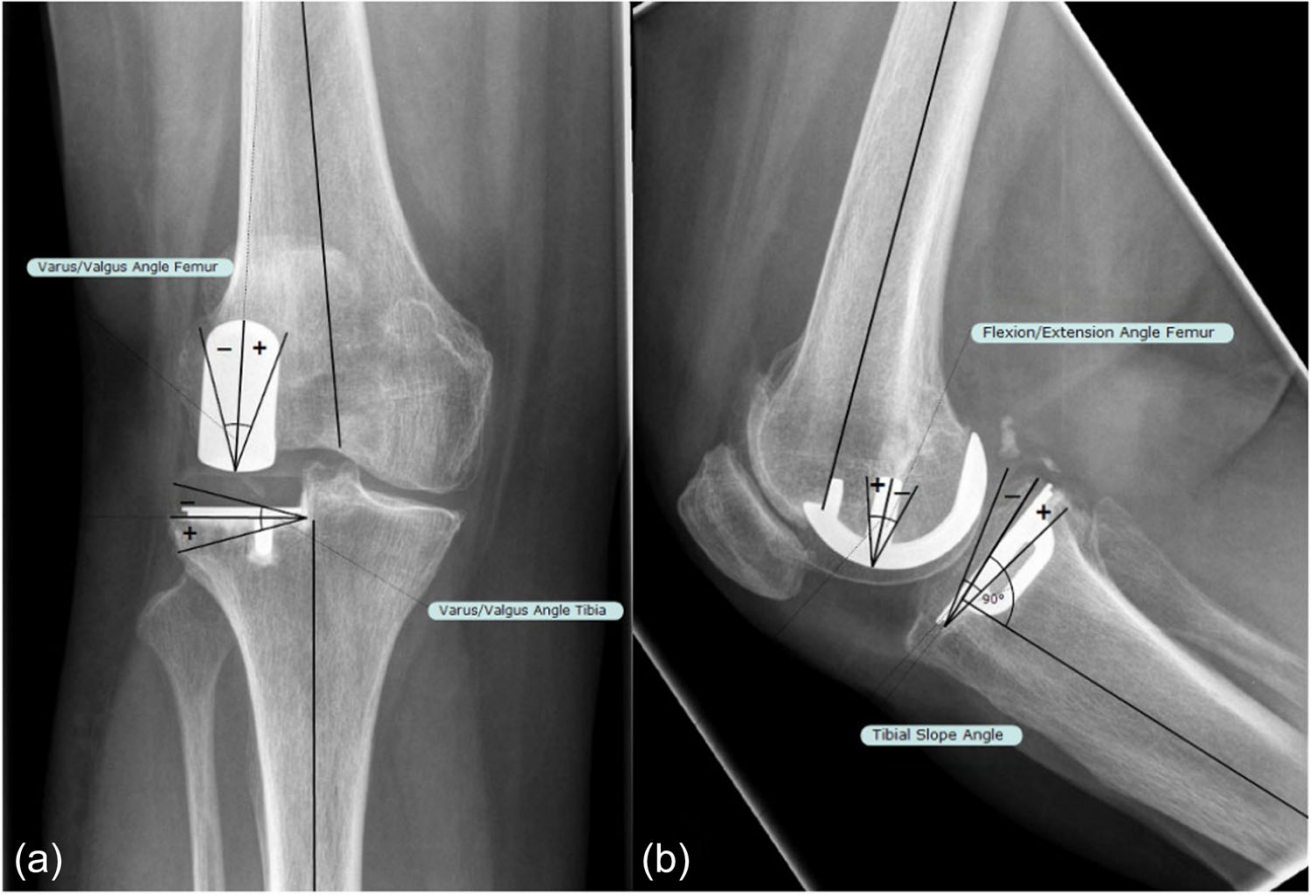
Radiographic assessment of femoral and tibial component alignment: (a) varus/valgus angle of femoral and tibial components on anteroposterior (AP) radiograph and (b) femoral flexion/extension angle and tibial slope angle on lateral radiographs. Positive values (+) indicate valgus and flexion for the femoral component, and valgus and posterior slope for the tibial component.

Radiographic analyses were independently performed by two observers using TraumaCad software (Brainlab, Munich, Germany) to assess coronal and sagittal alignment of the femoral and tibial components, referenced to the anatomical axes (varus/valgus angle of the femoral [VVA‐F] and tibial [VVA‐T] component, flexion/extension angle of the femoral component [FEA‐F], and tibial slope angle [TSA]; Figure 1). The anatomical axes of femur and tibia were determined by connecting the midpoints between bone cortices at two different levels in both planes using TraumaCad's centerline measurement tool. This tool was also used to determine the femoral component axis in both planes, while the tibial component axis was defined by referencing the inferior surface of the tibial component [9].

Inter‐ and intra‐observer reliability was assessed using intraclass correlation coefficients (ICC).

### Statistical analysis

All statistical analyses were performed using SPSS version 29.0 (SPSS Inc., Chicago, Illinois) and GraphPad Prism version 10.2.2 (GraphPad Software, San Diego, California). The Shapiro–Wilk test was used to assess normality, confirming that the data were not normally distributed. Therefore, Spearman's rank correlation coefficient was applied to assess associations between radiological and clinical parameters.

Where appropriate, subgroup analyses were conducted to further investigate relationships identified in the correlation analysis. Group differences were analysed using the Kruskal–Wallis test, followed by post hoc pairwise comparisons using the Mann–Whitney *U* test. To adjust for multiple comparisons, the Benjamini‐Hochberg procedure (False Discovery Rate correction) was applied, with a corrected *p* < 0.05 considered statistically significant.

Patients were further classified into ‘inlier’ or ‘outlier’ groups based on a ±1.5 standard deviation cutoff from the mean alignment. This cutoff was chosen due to the absence of established reference values for lateral UKA implant positioning and to ensure adequate subgroup sizes for robust statistical comparison. Clinical outcomes (OKS, FJS and ROM) were compared between these groups using the Mann–Whitney *U* test.

To exploratively identify optimal alignment ranges for femoral and tibial component positioning, patients were divided into quartiles based on the postoperative FJS. The mean values and standard deviations for femoral and tibial component angles were calculated specifically for patients achieving results in the highest quartile of the FJS. The FJS was chosen for the quartile‐based analysis due to its high sensitivity in detecting subtle differences in joint awareness and patient‐reported satisfaction, making it particularly suitable for identifying clinically relevant alignment ranges [18].

Multiple linear regression analysed the influence of patient‐specific factors (age, body mass index [BMI] and tibial inlay height) on OKS, FJS and ROM.

A priori power analysis was conducted to determine the required sample size, assuming a moderate effect size (*r* = 0.30), a significance level of 0.05, and a statistical power of 80%. The analysis, based on expected correlations involving the FJS, determined that a minimum of 89 patients was required to detect such an effect.

For all statistical tests, the significance level was set at *p* < 0.05.

## RESULTS

**FIGURE 2:**
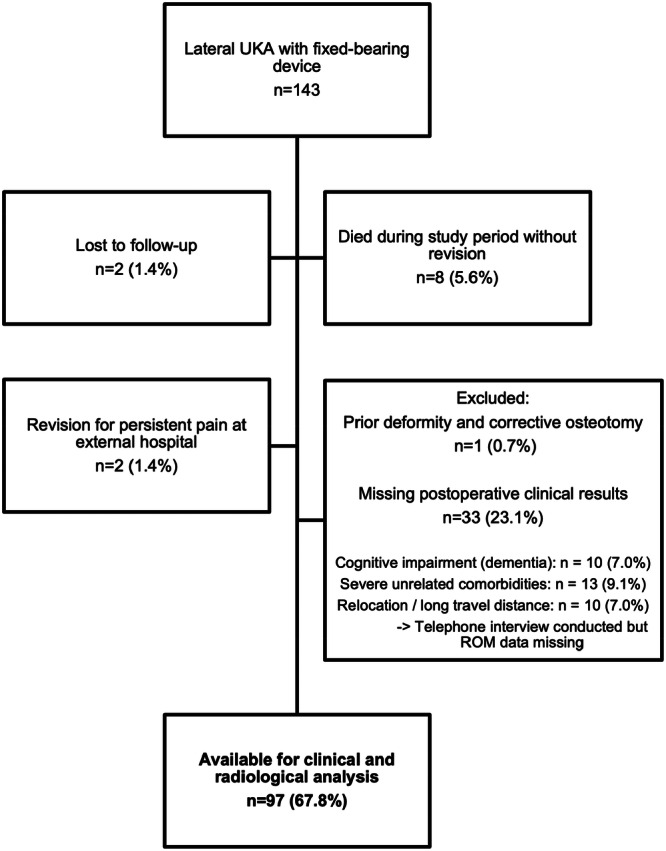
Flowchart illustrating the selection of the study population. ROM, range of motion; UKA, unicompartmental knee arthroplasty.

A total of 143 lateral UKAs were performed over the study period (Figure 2). Eight patients (5.6%) died during the study period for unrelated reason without revision surgery before their demise. Two patients (1.4%) were lost to follow‐up, one female patient (0.7%) was excluded due to prior corrective osteotomy and deformity and in 33 patients (23.1%) postoperative clinical data were partially missing as they did not attend for clinical follow‐up for various reasons (Figure 2). These patients or their relatives were contacted by telephone to confirm that no revision surgery had been performed during the study period. Two female patients (1.4%) were revised to TKA in external hospitals due to persistent pain without objective cause.

Therefore, 94 patients (97 UKA) were eligible for clinical and radiological analysis. Patients’ demographics are shown in Table 1.

**TABLE 1:** Demographics.

Demographics	
Patients (UKA)	94 (97)
Mean follow‐up in years ±SD (range)	3.4 ± 1.9 (1.0–8.5)
Mean age at time of surgery in years ±SD	64.5 ± 11.0 (28–84)
Sex (%)	Female: 67 (71.3%); male: 27 (28.7%)
Operated side (%)	Left: 31 (32.0%); right: 66 (68.0%)
Mean body mass index (kg/m^2^) ± SD (range)	27.7 ± 5.5 (17.7–49.1)
Tibial inlay height ±SD (range)	3.8 ± 0.9 (3–7)

Abbreviations: SD, standard deviation; UKA, unicompartmental knee arthroplasty.

### Clinical results

ROM improved from 119.7° ± 16.7° preoperatively to 134.0° ± 9.5° postoperatively (*p* < 0.001), and OKS increased from 27.4 ± 8.0 to 40.5 ± 7.0 (*p* < 0.001), while the mean postoperative FJS was 66.8 ± 28.1.

The ICC for intra‐ and inter‐observer reliability was good to excellent for all measurements (Table 2).

**TABLE 2:** Intra‐ and inter‐observer ICC for all measurements.

	Intra‐observer ICC	Inter‐observer ICC
Varus/valgus femoral component	0.97	0.93
Varus/valgus tibial component	0.90	0.89
Flexion/extension femoral component	0.99	0.98
Tibial slope angle	0.87	0.89

Abbreviation: ICC, interclass correlation coefficient.

The multiple linear regression analysis revealed no statistically significant associations between patient age, BMI, or tibial inlay height and postoperative clinical outcomes (OKS, FJS and ROM).

### Radiological results

The mean angle measurements as well as correlation analysis are demonstrated in Table 3.

**TABLE 3:** Mean values for all angle measurements with the correlation analysis for OKS, FJS and ROM.

	Mean ± SD	Spearman's rank correlation coefficient		
		OKS	FJS	ROM
VVA‐F	4.8° ± 5.6°	0.04	−0.02	−0.08
VVA‐T	−0.6° ± 2.6°	−0.11	−0.21^a^	−0.01
FEA‐F	6.6° ± 6.5°	0.07	0.07	0.02
TSA	7.4° ± 2.2°	−0.25^a^	−0.06	−0.12

*Note*: Positive values (+) indicate valgus and flexion for the femoral component, and valgus and posterior slope for the tibial component.

Abbreviations: FEA‐F, flexion/extension angle femoral component; FJS, forgotten joint score; OKS, oxford Knee score; ROM, range of motion; SD, standard deviation; TSA, tibial slope angle; VVA‐F, varus/valgus angle femoral component; VVA‐T, varus/valgus angle tibial component.

^a^ Indicating weak correlation.

The analysis revealed a weak negative correlation between the TSA and OKS (*r* = −0.25), as well as a weak negative correlation between the VVA‐T and FJS (*r* = −0.21). No relevant correlations were found between the remaining radiological parameters and the clinical outcomes.

Based on this finding, a subgroup analysis was performed to further investigate the relationship between VVA‐T and the FJS Score. Patients were categorised into three groups: varus (VVA‐T < 0°; *n* = 47), neutral (VVA‐T = 0°; *n* = 22), and valgus (VVA‐T > 0°; *n* = 28). The mean postoperative FJS was highest in the varus group (74.0 ± 27.6), followed by the valgus group (62.3 ± 25.2), and lowest in the neutral group (57.3 ± 29.9). A Kruskal–Wallis test showed a significant difference (*p* = 0.021). Post hoc Mann–Whitney *U* tests confirmed that FJS was significantly higher in the varus group compared to neutral (*p* = 0.012) and valgus (*p* = 0.049). After Benjamini–Hochberg correction, the difference between varus and neutral remained significant (*p* = 0.035), while varus versus valgus lost significance (*p* = 0.073). No significant difference was found between neutral and valgus (*p* = 0.577).

Additionally, a subgroup analysis examined TSA and OKS (*r* = −0.25). Patients were also categorised into three groups: flat (TSA < 7°; *n* = 31), neutral (TSA = 7°; *n* = 21) and steep (TSA > 7°; *n* = 45). The mean postoperative OKS was highest in the flat group (41.8 ± 7.7), followed by the neutral group (41.2 ± 5.5), and lowest in the steep group (39.2 ± 7.1). A Kruskal–Wallis test showed no significant difference (*p* = 0.069).

### Outlier analysis

**FIGURE 3:**
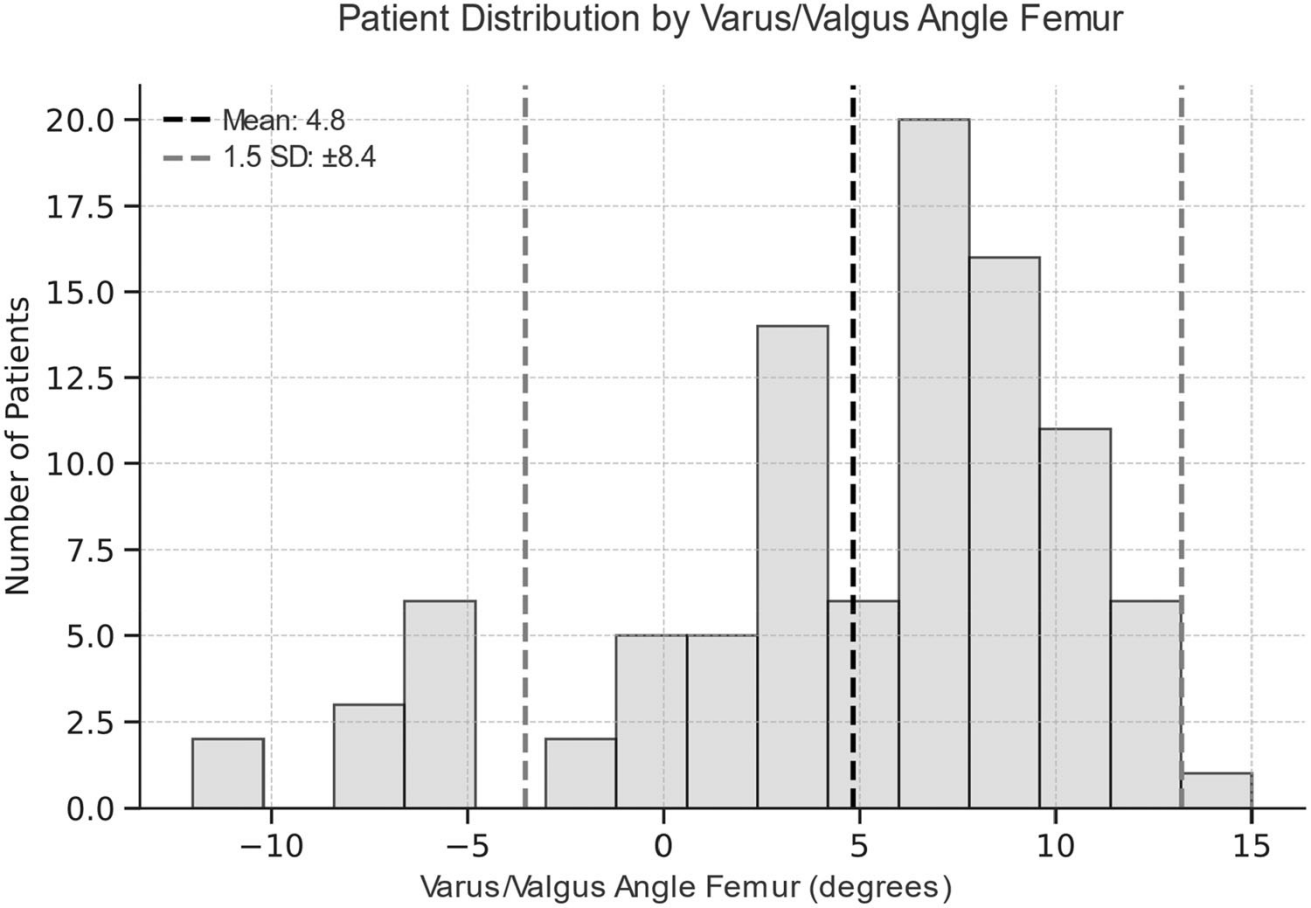
Histogram illustrating the distribution of varus/valgus angle of the femoral component across the patient population. The dashed black line indicates the mean angle (4.8°), and the dashed grey lines represent ±1.5 standard deviation (SD) defining the thresholds for outliers. Positive (+) values indicate valgus, negative (−) values indicate varus.

**FIGURE 4:**
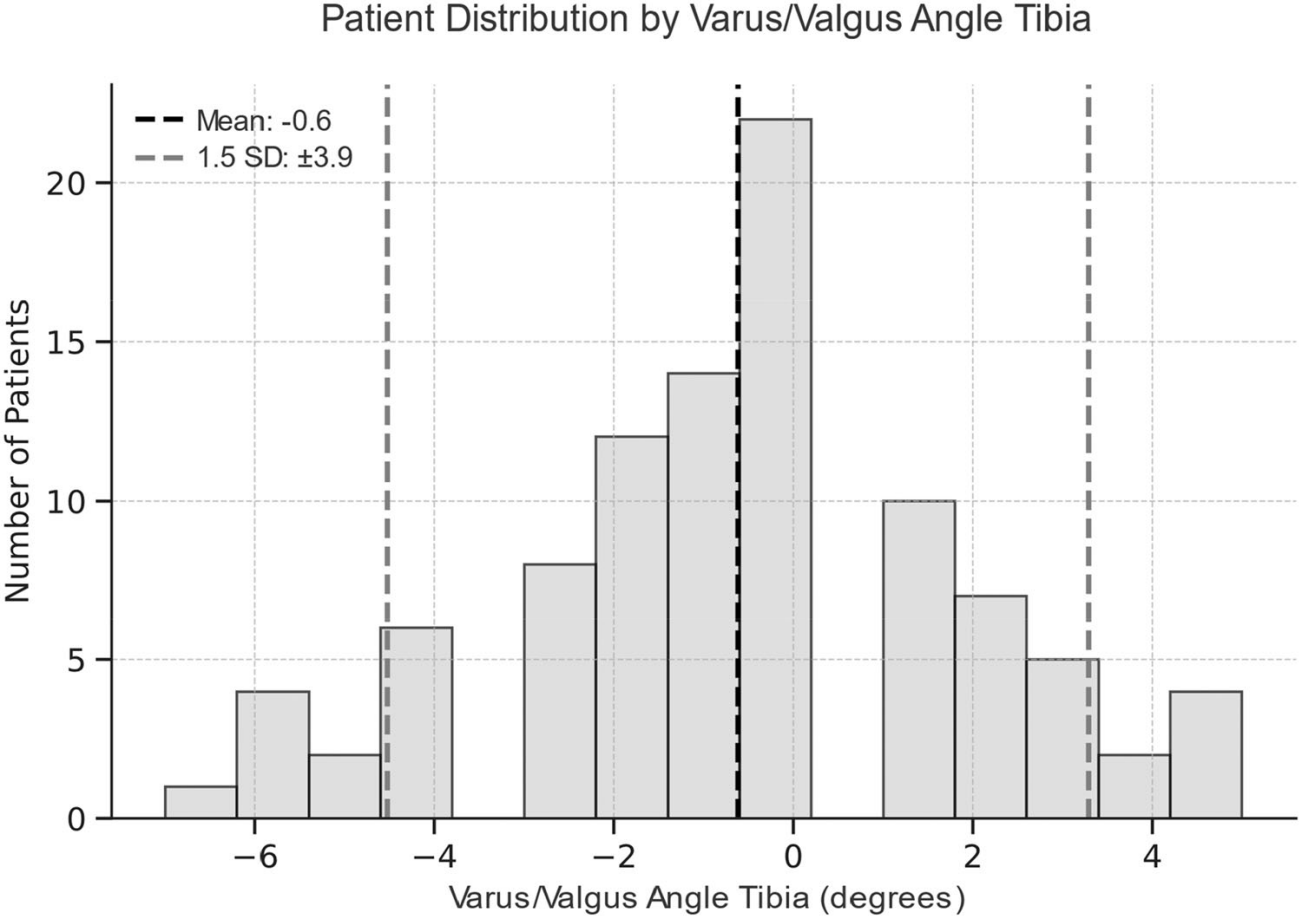
Histogram illustrating the distribution of varus/valgus angle of the tibial component across the patient population. The dashed black line indicates the mean angle (−0.6°), and the dashed grey lines represent ±1.5 standard deviation (SD) defining the thresholds for outliers. Positive (+) values indicate valgus, negative (−) values indicate varus.

**FIGURE 5:**
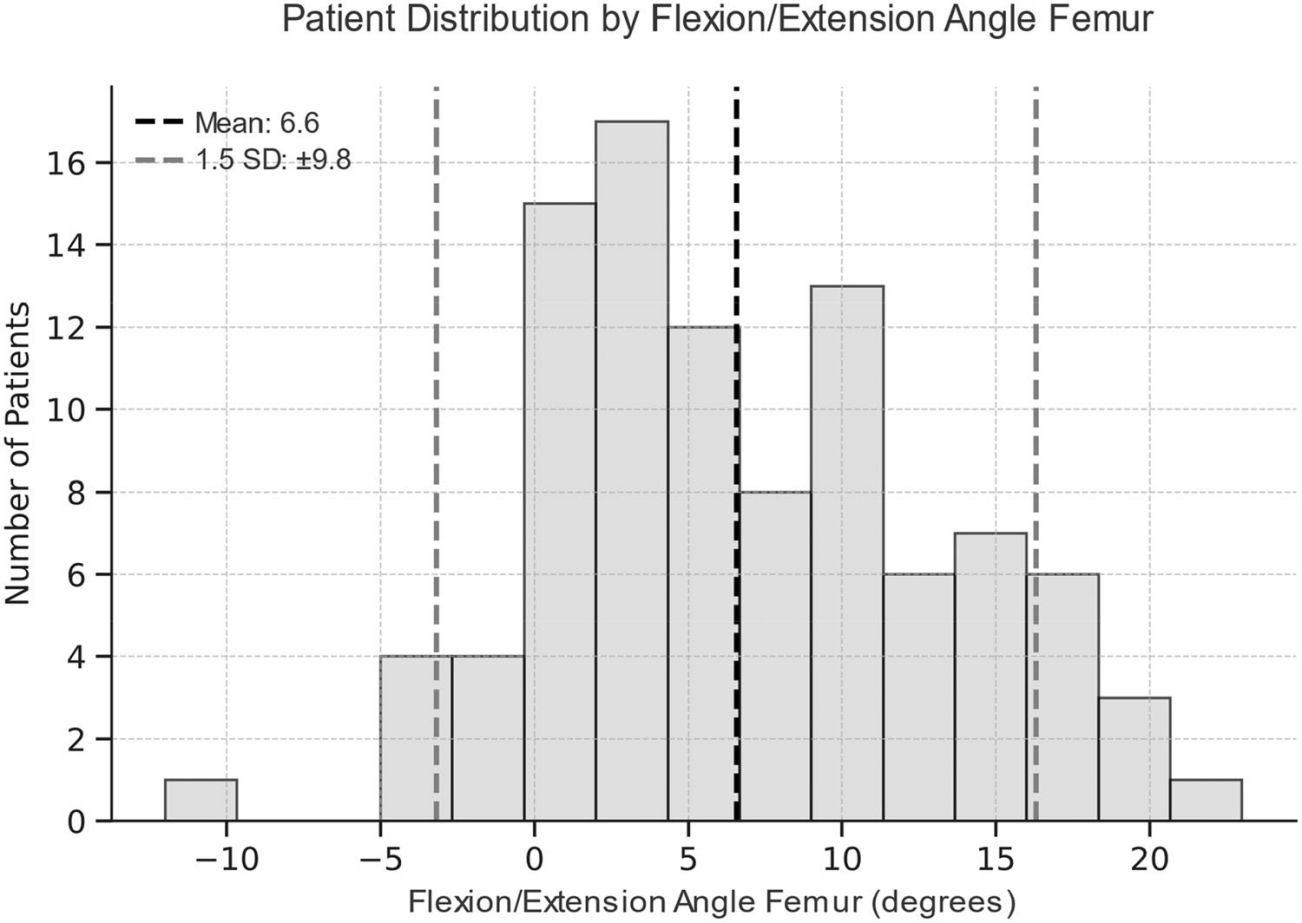
Histogram illustrating the distribution of flexion/extension angle of the femoral component across the patient population. The dashed black line indicates the mean angle (6.6°), and the dashed grey lines represent ±1.5 standard deviation (SD) defining the thresholds for outliers. Positive (+) values indicate flexion, negative (−) values indicate extension.

**FIGURE 6:**
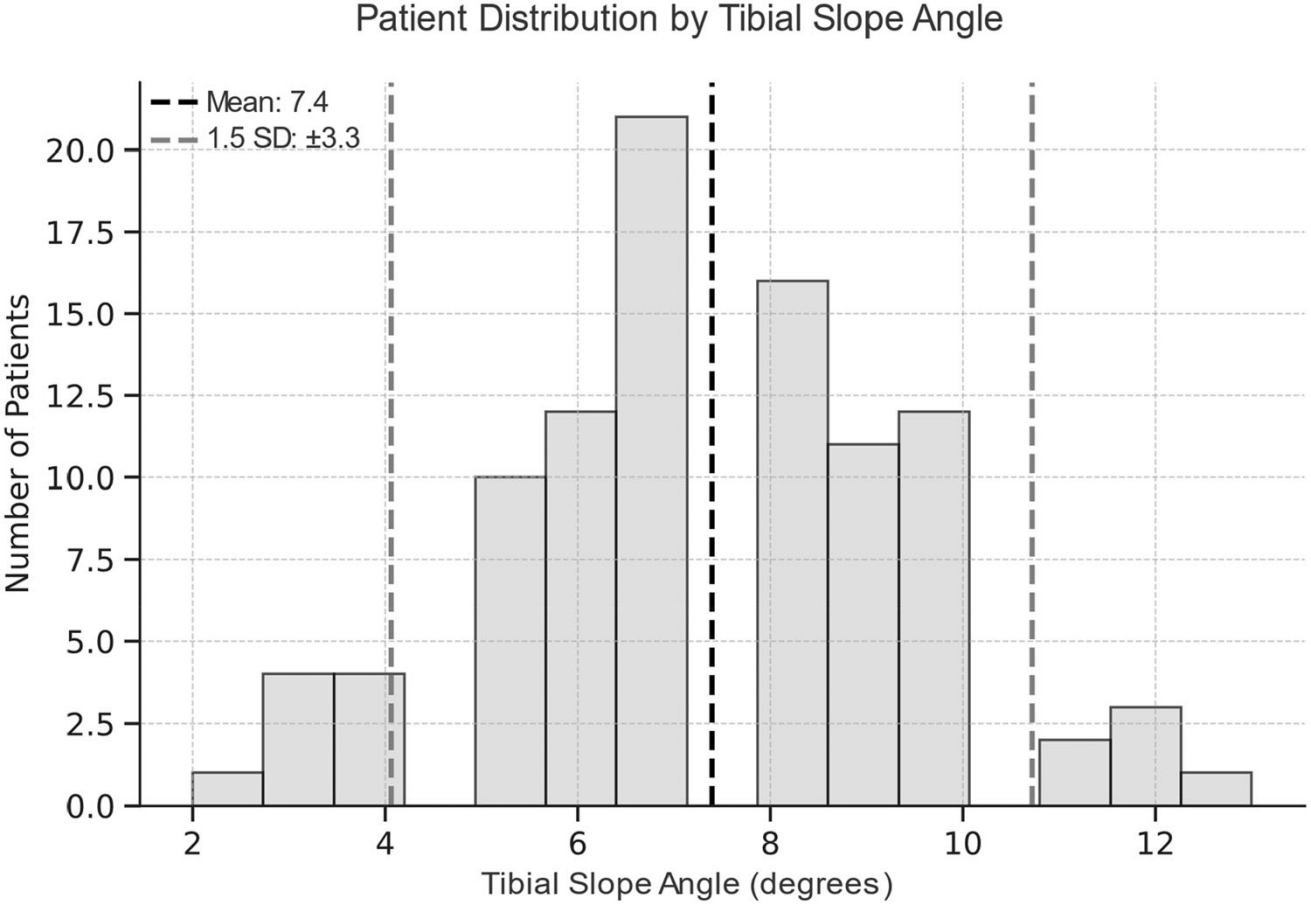
Histogram illustrating the distribution of tibial slope angles across the patient population. The dashed black line indicates the mean slope angle (7.4°), and the dashed grey lines represent ±1.5 standard deviation (SD) defining the thresholds for outliers.

The group comparison between outlier and inlier revealed a significant higher FJS for inlier in VVA‐T angle (Table 4). All other comparison showed no significant differences. All thresholds for defining the groups are illustrated in Figures 3, 4, 5, 6.

**TABLE 4:** Comparison of clinical results between outlier and inlier for each angle based on a ±1.5 standard deviation cutoff from the mean alignment.

		Inlier group	Outlier group	*p* value
OKS ± SD (*n*)	VVA‐F	40.3 ± 7.1 (85)	41.9 ± 6.4 (12)	0.54
	VVA‐T	40.8 ± 7.0 (84)	38.6 ± 7.2 (13)	0.23
	FEA‐F	40.7 ± 6.9 (87)	39.0 ± 8.3 (10)	0.54
	TSA	40.9 ± 6.6 (82)	38.1 ± 9.0 (15)	0.39
FJS ± SD (*n*)	VVA‐F	65.6 ± 29.1 (85)	75.4 ± 17.8 (12)	0.42
	VVA‐T	69.1 ± 28.5 (84)	52.1 ± 20.7 (13)	0.04^a^
	FEA‐F	68.7 ± 27.5 (87)	50.4 ± 29.8 (10)	0.06
	TSA	67.0 ± 28.2 (82)	66.1 ± 29.0 (15)	0.93
ROM ± SD (*n*)	VVA‐F	134.1 ± 9.2 (85)	133.3 ± 12.1 (12)	0.79
	VVA‐T	134.2 ± 9.7 (84)	132.7 ± 8.6 (13)	0.63
	FEA‐F	134.5 ± 9.6 (87)	131.0 ± 8.4 (10)	0.25
	TSA	133.6 ± 10.0 (82)	136.3 ± 6.4 (15)	0.27

Abbreviations: FEA‐F, flexion/extension angle femoral component; FJS, forgotten joint score; OKS, Oxford Knee score; ROM, range of motion; SD, standard deviation; TSA, tibial slope angle; VVA‐F, varus/valgus angle femoral component; VVA‐T, varus/valgus angle tibial component.

^a^ Indicating significance.

### Quartile analysis

**FIGURE 7:**
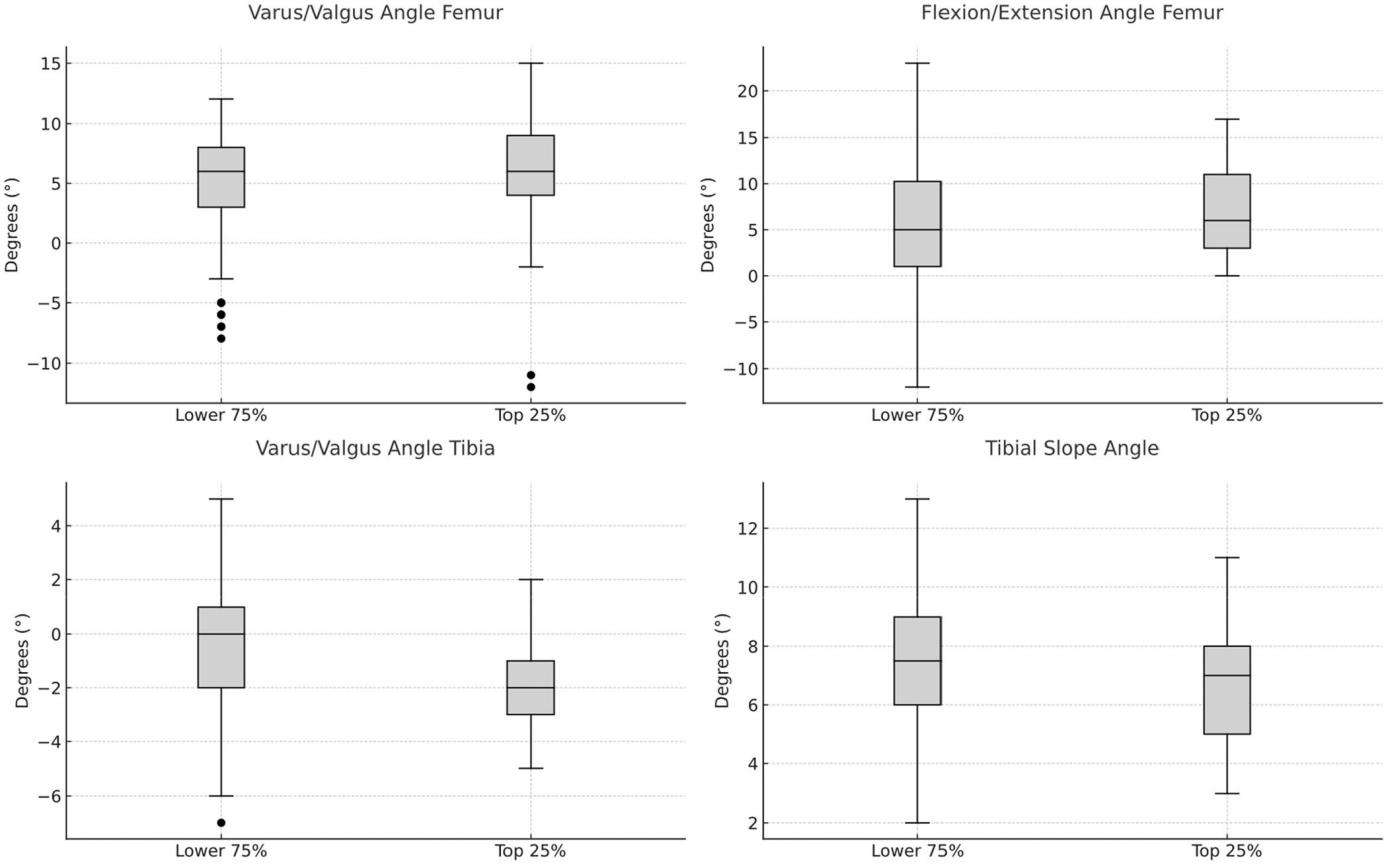
Boxplots illustrating the distribution of alignment angles of femoral and tibial components for patients categorised into two groups based on postoperative Forgotten Joint Score (FJS). ‘Top 25%’ refers to patients scoring in the highest quartile of the FJS (indicating better joint awareness and satisfaction), whereas ‘Lower 75%’ represents patients below this threshold. The angles analysed include the femoral varus/valgus angle (top left), femoral flexion/extension angle (top right), tibial varus/valgus angle (bottom left), and tibial slope angle (bottom right). Boxes indicate the interquartile range (IQR), horizontal lines indicate the median, whiskers represent 1.5 times the IQR, and points depict outliers.

The exploratory quartile analysis based on the top‐performing patients according to the FJS identified component alignment ranges associated with optimal clinical outcome (Table 5). Patients in the top 25% group had an FJS of ≥91.7 points. While there was a significant difference between the two groups in the VVA‐T (*p* = 0.01), no significant differences were observed for the other angles (VVA‐F: *p* = 0.79; FEA‐F: *p* = 0.27; TSA: *p* = 0.11; Figure 7).

**Table 5:** Recommended range for component alignment based on the top‐performing patients (*n* = 29, highest quartile) according to the FJS.

	Mean ± SD	Recommended range
VVA‐F	5.0° ± 6.2°	−1° to 11°
VVA‐T	−1.1° ± 2.2°	−3° to 1°
FEA‐F	7.5° ± 5.4°	2°–13°
TSA	6.8° ± 2.2°	5°–9°

*Note*: Positive values (+) indicate valgus and flexion for the femoral component, and valgus and posterior slope for the tibial component.

Abbreviations: FEA‐F, flexion/extension angle femoral component; FJS, forgotten joint score; SD, standard deviation; TSA, tibial slope angle; VVA‐F, varus/valgus angle femoral component; VVA‐T, varus/valgus angle tibial component.

## DISCUSSION

The main finding of this study is that component alignment within the observed range was not significantly associated with clinical outcomes after lateral UKA using a fixed‐bearing design, with the exception of joint awareness. Patients with a varus‐aligned tibial component reported significantly higher FJS values, suggesting a possible relationship between slight varus alignment and improved joint perception. The data suggest a recommended range for the VVA‐T between 3° varus and 1° valgus, indicating that surgeons may favour a slight varus rather than valgus alignment of the tibia. For all other measurements, over 85% of knees were positioned within a broad range of ±1.5 SD from the mean value without any impairment of clinical outcomes. Since this is the largest exploratory study analysing the association between component alignment and clinical results in lateral UKA, the authors propose the following target alignment ranges, referenced to the anatomical axes of the femur and tibia, as safe: VVA‐F: 3° varus to 13° valgus, VVA‐T: 3° varus to 1° valgus, FEA‐F: 3.5° extension to 16.5° flexion and TSA: 4.5°–10.5°.

It remains unclear to what extent component alignments outside the observed range are associated with poorer outcomes. Given the exploratory nature of this analysis and the limited follow‐up period, these findings should be interpreted as preliminary and hypothesis‐generating rather than conclusive.

The findings of the present study regarding the importance of tibial component alignment align with some studies on medial UKA, which have implicated tibial component alignment as a factor influencing UKA survival [4, 6, 12]. In UKA, malalignment of the tibial component influences parameters such as joint line obliquity (JLO) and joint line convergence angle (JLCA) and is less forgiving than malalignment of the femoral component, which compensates for a broader range due to its spherical design [27]. While JLO and JLCA directly influence knee joint kinematics, Sahbat et al. [38] found that postoperative changes in coronal knee alignment and JLO had no effect on clinical outcomes in medial UKA.

One possible explanation for the improved joint awareness in patients with a varus‐aligned tibial component in lateral UKA may be the restoration of the pre‐arthritic JLO, as the majority of knees exhibit an apex distal configuration according to the coronal plane alignment of the knee classification [24]. Since UKA aims to replicate the pre‐arthritic state and prioritise soft tissue balance, a varus‐aligned tibial component may help achieve this goal in most patients [27]. This hypothesis, however, requires confirmation through future studies specifically addressing the influence of tibial alignment on JLO and patient‐reported outcomes in lateral UKA.

Slaven et al. [41] demonstrated in a large cohort of 3351 medial UKA cases that limb alignment was more critical than component alignment in terms of revision risk and clinical outcomes. While in TKA, component alignment is strongly associated with limb alignment, in UKA, component thickness rather than the component alignment plays a key role, which may explain why UKA is more forgiving in cases of component malalignment [10]. In this study, the tibial inlay height showed no association with clinical results.

Most previous studies on the effect of component alignment in medial UKA with both mobile‐bearing and fixed‐bearing designs have found no significant correlation with clinical outcomes [5, 9, 10]. Interestingly, no known study analysing component alignment in UKA has used the FJS for clinical assessment. The FJS is recognised for its high sensitivity in detecting subtle differences in joint awareness while exhibiting low ceiling effects [18]. Consequently, previous studies may not have identified small differences in functional outcomes.

The majority of previous studies on this topic were limited to short‐ and mid‐term results, whereas a recent study by Khow et al. demonstrated poorer functional outcomes after 10 years, as measured by the OKS, in patients with a varus malalignment of the femoral component (>3° varus relative to the mechanical leg axis). Notably, this difference was not observed in the short‐term [16]. The authors attributed this to potentially higher contact stress compared to a neutrally aligned femoral component, leading to increased wear over time [13, 16]. However, it is well established that contact areas are determined by the alignment of both implants, meaning that loading patterns cannot be attributed solely to the alignment of a single component [45]. To minimise localised contact stress, Kang et al. [14] recommended a combined neutral alignment of both components based on findings from a finite element study. The extent to which these findings are transferable to patients with lateral UKA remains questionable due to the different kinematic characteristics [42]. Furthermore, as this study is based on short‐ to mid‐term data, no conclusions can be drawn regarding long‐term outcomes.

UKA is considered a technically demanding procedure, where surgical experience is strongly associated with clinical results and revision risk [21, 35]. Implant positioning in lateral UKA can be particularly challenging due to the limited exposure provided by the lateral approach [42]. While the surgical instrumentation of the implant used enables precise implantation in medial UKA, the broad range of component alignments observed in this study suggests greater difficulties in achieving optimal positioning in lateral UKA [31]. Kazarian et al. reported a wide distribution of radiographic outliers in 253 patients undergoing fixed‐bearing medial UKA performed by two experienced surgeons with only 11.9% of patients having all components aligned within proposed range [15]. To mitigate component malalignment caused by technical errors, robotic‐assisted implantation can be utilised, as it significantly improves accuracy [2, 8, 19]. However, to date, no clinical benefit in terms of implant survivorship or patient outcomes has been demonstrated with the use of robotic assistance [25, 28, 36, 47].

This study has several limitations. First, its retrospective design introduces a potential selection bias, as not all patients were available for analysis. Second, the follow‐up period was limited to the short‐ to mid‐term, preventing conclusions regarding long‐term effects. In particular, the impact of component alignment on revision risk due to wear or implant loosening may be underrepresented. However, this study primarily focused on clinical outcomes, and a survival analysis was not statistically meaningful due to the low revision rate.

Third, while the observed relationships between component alignment and postoperative outcomes may inform further prospective studies, no conclusions regarding causality can be drawn from this retrospective analysis.

Finally, other implant‐related factors such as component overhang, rotational alignment, tibial coverage, limb alignment, JLO and JLCA were not assessed in this study, as the primary focus was on component alignment in the coronal and sagittal planes based on standard postoperative radiographs These variables may influence clinical outcomes and should be addressed in future analyses. While short‐film radiographs of the knee have been shown to provide accurate measurements of component alignment, postoperative changes in limb alignment, JLO and JLCA are difficult to assess using this method [40, 43, 46]. One study proposed a formula to estimate the postoperative HKA based on preoperative whole‐leg radiographs and demonstrated its accuracy. This approach may be useful in future studies with long‐term follow‐up to evaluate the potential influence of limb alignment on implant survivorship [33].

## CONCLUSION

Although previous studies have addressed component alignment in UKA, evidence specifically related to lateral fixed‐bearing UKA remains limited. The present study contributes to this underexplored area by providing preliminary data suggesting that slight varus alignment of the tibial component may be associated with improved clinical outcomes in lateral UKA.

## AUTHOR CONTRIBUTIONS

All authors contributed to the study conception and design. Mustafa Hariri contributed to acquisition, analysis and interpretation of data and drafted the manuscript and figures. Timo A. Nees, Tilman Walker, Paul Mick, Tobias Reiner and Kevin‐Arno Koch performed data collection and revised the manuscript critically. Johannes Weishorn performed the statistical analysis and revised the manuscript critically. Tilman Walker participated in the study design and helped to draft the manuscript. Raphael Trefzer drafted the manuscript and revised it critically.

## CONFLICT OF INTEREST STATEMENT

All authors declare that they have no competing interests.

## ETHICS STATEMENT

Ethical approval was obtained by the institutional review boards of the University of Heidelberg (S‐079/2023) and the study was conducted in accordance with the Helsinki Declaration of 1975, as revised in 2013. Informed consent was obtained from all participants included in the study.

## Data Availability

The data will be available upon reasonable request.
